# Association of <em>TGFβ2</em> gene polymorphism with growth performance and meat quality traits in Kampung Unggul Balitbangtan chickens under multienzyme supplementation

**DOI:** 10.14202/vetworld.2026.3125-3137

**Published:** 2026-07-20

**Authors:** Siti Rani Ayuti, Mirni Lamid, Mohammad Anam Al Arif, Sunaryo Hadi Warsito, Eun Joong Kim, Sangsu Shin, Latifah Latifah, Aswin Rafif Khairullah

**Affiliations:** 1 Laboratory of Biochemistry, Faculty of Veterinary Medicine, Universitas Syiah Kuala, Jl. Tgk. Hasan Krueng Kalee No.4, Kopelma Darussalam, Banda Aceh 23111, Aceh, Indonesia.; 2 Division of Animal Husbandry, Faculty of Veterinary Medicine, Universitas Airlangga, Jl. Dr. Ir. H. Soekarno, Kampus C Mulyorejo, Surabaya 60115, East Java, Indonesia.; 3 Department of Animal Science and Biotechnology, Kyungpook National University, Sangju 37224, Republic of Korea.; 4 Research Center for Animal Husbandry, National Research and Innovation Agency (BRIN), Jl. Raya Bogor Km. 46 Cibinong, Bogor 16911, West Java, Indonesia.; 5 Research Center for Veterinary Science, National Research and Innovation Agency (BRIN), Jl. Raya Bogor Km. 46 Cibinong, Bogor 16911, West Java, Indonesia.

**Keywords:** body weight, feed conversion ratio, Kampung Unggul Balitbangtan chicken, marker-assisted selection, meat quality, multienzyme supplementation, TGFβ2 gene, tropical poultry genetics

## Abstract

**Background and Aim:**

Improving growth performance and meat quality is a major objective in genetic improvement programs for indigenous poultry breeds. Kampung Unggul Balitbangtan (KUB) chickens are an improved Indonesian native breed with considerable potential for sustainable tropical poultry production. *Transforming growth factor-beta 2 (TGFβ2)* plays an important role in regulating skeletal muscle development, cell proliferation, and tissue remodeling; however, its genetic variation and association with economically important traits in KUB chickens remain unknown. This study aimed to characterize *TGFβ2* gene polymorphisms and evaluate their associations with growth performance and meat quality traits in KUB chickens receiving dietary multienzyme supplementation.

**Materials and Methods:**

Twenty-five one-day-old male KUB chickens were assigned to five dietary treatments containing different combinations of phytase and protease and reared for 90 days. Growth performance was assessed using feed intake (FI), body weight (BW), feed conversion ratio (FCR), and carcass weight (CW). Meat quality traits, including pH, texture, cooking loss (CL), water holding capacity, and color characteristics (L*, a*, and b*), were evaluated after slaughter. Genomic DNA extracted from muscle tissue was analyzed using polymerase chain reaction-restriction fragment length polymorphism and DNA sequencing to identify *TGFβ2* polymorphisms. Functional enrichment of differentially expressed genes was investigated using Gene Ontology and Kyoto Encyclopedia of Genes and Genomes pathway analyses.

**Results:**

Three *TGFβ2* genotypes (CC, TC, and TT) were identified, with allele frequencies of 0.48 (C) and 0.52 (T). The *TGFβ2* genotype was significantly associated with BW, CW, and FCR (p < 0.05), whereas FI was unaffected. Multienzyme supplementation significantly reduced CL, improved water holding capacity, and enhanced meat color characteristics without affecting pH or texture. Two exon single nucleotide polymorphisms, c.103C>T and c.99G>A, were identified, representing novel variants in KUB chickens. Functional enrichment analyses demonstrated significant involvement of extracellular matrix-receptor interaction, metabolic pathways, and biological processes related to muscle development and cell proliferation, supporting a nutrigenetic interaction between *TGFβ2* polymorphism and enzyme supplementation.

**Conclusion:**

This study provides the first characterization of *TGFβ2* polymorphisms in KUB chickens and demonstrates their association with growth performance and meat quality traits under multienzyme supplementation. The identified polymorphisms represent promising molecular markers for marker-assisted selection and precision nutrition strategies to improve productivity in indigenous tropical chicken populations.

## INTRODUCTION

Chickens are among the most important poultry species supporting food security and rural livelihoods in Indonesia, particularly for smallholder farmers who rely on them as a primary source of animal protein and household income [1]. Among the diverse native chicken populations, the Kampung Unggul Balitbangtan (KUB) chicken, developed by the Indonesian Agency for Agricultural Research and Development (Balitbangtan), has emerged as a genetically improved line with enhanced productivity, adaptability, and resilience to tropical environmental conditions [2]. KUB chickens were selectively bred from local village chickens through a systematic genetic improvement program to optimize egg production, growth rate, and feed efficiency while retaining the robust adaptive and behavioral characteristics of indigenous breeds [3]. Continuous genetic improvement is essential to sustain and enhance the performance of KUB chickens, given their dual-purpose role in egg and meat production and their socio-economic importance to smallholder poultry systems [4]. Their superior meat flavor, disease tolerance, and ability to thrive under high-temperature environments highlight their potential as a strategic genetic resource for sustainable poultry production in tropical regions.

Genetic factors governing growth and carcass traits are heritable and thus highly valuable for breeding programs aimed at enhancing productivity [5]. Investigating gene polymorphisms and their associations with economic traits offers a powerful approach for identifying candidate alleles that can be utilized as molecular markers in marker-assisted selection (MAS) programs [6]. Single-nucleotide polymorphisms (SNPs), in particular, provide high-resolution markers for studying genetic variation, yet their utility requires validation across diverse populations and breeds [7]. Among candidate genes associated with growth and muscle development, the transforming growth factor-beta 2 (*TGFβ2*) gene, located on chromosome 3, is recognized as a key regulator of cellular proliferation, differentiation, tissue remodeling, and immune response modulation [8, 9]. Previous studies have linked *TGFβ2* gene polymorphisms to growth performance and meat quality traits in several livestock species, including cattle, pigs, and commercial chicken lines [10]. In chickens, *TGFβ2* polymorphisms have been associated with growth and production traits in commercial broilers, layer lines, and several indigenous breeds. However, these findings cannot be directly extrapolated to genetically distinct native-derived populations, as breed-specific genetic architecture and selection history may influence allelic distribution and phenotypic effects [11]. To date, no study has characterized *TGFβ2* genetic variation or evaluated its association with economic traits in the selectively bred KUB chicken, Indonesia’s improved native dual-purpose breed developed for tropical smallholder production systems.

Although *TGFβ2* gene polymorphisms have been investigated in commercial broiler and layer lines as well as some indigenous chicken breeds, information on their occurrence, distribution, and functional significance in selectively improved native breeds such as KUB chickens is lacking. Previous studies have primarily examined associations with growth traits under standard feeding conditions, with limited attention to potential interactions between *TGFβ2* variants and nutritional interventions such as multienzyme supplementation. Furthermore, integrated evaluations of *TGFβ2* polymorphisms on both growth performance and meat quality traits in tropical smallholder production contexts remain scarce. This gap limits the application of MAS programs tailored to the genetic resources of Indonesian native poultry.

Therefore, the objective of this study was to characterize *TGFβ2* gene polymorphisms in KUB chickens and to evaluate their associations with growth performance and meat quality traits under varying levels of dietary multienzyme supplementation. This research is expected to provide novel molecular markers and nutrigenetic insights that support precision breeding and feeding strategies for sustainable productivity in indigenous tropical chicken populations.

## MATERIALS AND METHODS

### Ethical approval

The experimental protocol was reviewed and approved by the Animal Care and Use Ethics Committee, Faculty of Veterinary Medicine, Universitas Airlangga, Indonesia (Approval No. 1.KEH.039.03.2024). All procedures involving KUB chickens were conducted in accordance with the Indonesian national guidelines for the ethical care and use of animals in research, the institutional animal welfare requirements of Universitas Airlangga, and the principles outlined in the ARRIVE 2.0 guidelines.

Throughout the 90-day experimental period, the birds were housed under appropriate environmental, hygienic, and husbandry conditions, with unrestricted access to clean drinking water and nutritionally adequate feed in accordance with their assigned dietary treatments. The chickens were observed regularly for general health, behavior, feed and water consumption, mobility, signs of pain or distress, injury, and adverse responses to the experimental diets. All handling, weighing, sampling, and husbandry procedures were performed by trained personnel using methods designed to minimize fear, discomfort, restraint time, and unnecessary stress.

Predefined humane considerations were applied throughout the experiment. Any bird showing persistent, severe distress; inability to access feed or water; marked loss of body condition; serious injury; or clinical deterioration that could not be promptly alleviated was to be evaluated by qualified veterinary personnel and either removed from the experiment or, when necessary, humanely euthanized. At the end of the feeding trial, the chickens were slaughtered using the humane procedure authorized in the approved institutional protocol. Tissue collection was performed only after confirmation of death. Muscle samples were collected aseptically for meat quality assessment and molecular analysis, immediately processed or preserved as required, and stored under appropriate conditions.

The number of birds was limited to the minimum considered necessary to address the study objectives while permitting the planned statistical analyses. All reasonable measures were taken to refine the experimental procedures, reduce bird use, and minimize pain and distress. No procedures were performed solely for purposes unrelated to the approved scientific objectives.

### Study period and location

This study was conducted from June 5 to September 6, 2024 at the Faculty of Veterinary Medicine, Airlangga University.

### Study design

A total of 25 one-day-old male KUB chicks were randomly assigned to five dietary treatments (n = 5 per treatment). The experiment followed a 5 × 3 factorial arrangements comprising five dietary treatments and three *TGFβ2* genotypes (CC, TC, and TT), with genotypes determined post hoc by molecular analysis. The dietary treatments were: F0P0 (control, no enzymes), F4P0 (400 mg/kg phytase), F4P3 (400 mg/kg phytase + 300 mg/kg protease), F4P5 (400 mg/kg phytase + 500 mg/kg protease), and F4P7 (400 mg/kg phytase + 700 mg/kg protease). Enzyme inclusion levels were selected based on commercial recommendations and previous studies in native chickens. Birds were housed individually and fed *ad libitum* twice daily, with free access to water. All procedures were approved by the institutional ethics committee and conducted in accordance with animal welfare guidelines.

### Evaluation of performance

Body weight (BW) was recorded individually using a digital hanging scale with an accuracy of 0.01 g at day 1 and at the end of the experimental period (90 days of age). Birds were weighed weekly to monitor growth performance. Average daily gain (ADG) was calculated using the following formula: ADG (g/day) = (IBW − BW) / days, where BW represents BW (g). Feed intake (FI) was calculated as the difference between feed offered and feed refused for each bird, adjusted for any feed spillage. Daily FI (DFI) was determined by dividing total feed consumption by the number of feeding days. Feed conversion ratio (FCR) was calculated as: FCR = Total FI (g) / Total BW gain (g)

### Meat quality analysis

Meat quality parameters were analyzed including water-holding capacity (WHC), pH, texture, and cooking loss (CL), which were measured using 5 g of raw meat samples as the initial weight, with two replicates for each treatment. Meat color was evaluated using a colorimeter (12 mm aperture, U 59730-30, Cole-Parmer International Inc., Pittsford, NY, USA) on first use at the same measurement point for each sample. Meat color characteristics were then expressed in Lightness (L*), Redness (a*), and Yellowness (b*) values [12].

### Meat sampling and DNA isolation

Muscle samples weighing approximately 0.5-1.0 g of lean tissue were taken and placed into sterile cryovial tubes. Following instant flash freezing in liquid nitrogen, the samples were stored at −80 °C until DNA extraction and additional molecular analysis. The specific primer sequences used for the chicken *TGFβ2* target gene KUB (GenBank accession no. X58071) were as follows: forward primer, 5’-GCC ATA GGT TCA GTG CAA G-3’; reverse primer, 5’-TGA CAG AAG CTC TCA AGC C-3’. The manufacturer's instructions (Promega, Madison, WI, USA) on first use were followed to generate the polymerase chain reaction (PCR) reaction mixture, which included 12.5 μL of master mix, 1 μL of forward primer, 1 μL of reverse primer, 3 μL of DNA template, and 7.5 μL of distilled water. The ideal conditions for amplification were 5 min of initial denaturation at 95°C, 30 cycles of denaturation at 95°C for 30 seconds, annealing at 54°C for 30 seconds, extension at 72°C for 30 seconds, and a final extension at 72°C for 7 min.

### PCR-Restriction fragment length polymorphism (RFLP) analysis

PCR-RFLP analysis involved digesting 20 μL of PCR product with 1 μL of the restriction enzyme AluI for 3 h at 37°C. Following digestion, the pieces were separated using 1.5% agarose gel electrophoresis, stained with ethidium bromide, and examined under an ultraviolet lamp. Additionally, 20 μL of the PCR product was sent to Macrogen (Seoul, Korea) for sequencing analysis on first use [13].

### Screening and functional analysis of differentially expressed genes (DEGs)

DEGs were subjected to hierarchical clustering analysis using the Gplots package in the R software environment (R Foundation for Statistical Computing, Vienna, Austria). Following gene identification, STRING functional protein association networks were used to build a regulatory enrichment network, and Cytoscape was used to visualize it. The web-based program DAVID was used for Gene Ontology (GO) enrichment analysis, and the significance threshold for relevant GO categories was set at p < 0.05. Additionally, KOBAS 3.0 was used to conduct Kyoto Encyclopedia of Genes and Genomes (KEGG) pathway enrichment analysis [14].

### Statistical analysis

The Benjamini-Hochberg method was used to calculate adjusted p-values for DEG analysis and KEGG pathway enrichment. For growth performance and meat quality traits, data were analyzed using a two-way analysis of variance to evaluate the main effects of *TGFβ2* genotype and enzyme supplementation level, as well as their interaction (genotype × enzyme level). When significant effects were detected (p < 0.05), treatment means were compared using Duncan’s Multiple Range Test. All statistical analyses were performed using SPSS version 20.0. Results are presented as mean ± standard deviation (SD).

## RESULTS

### Functional and pathway enrichment analysis of target genes

**Figure 1:**
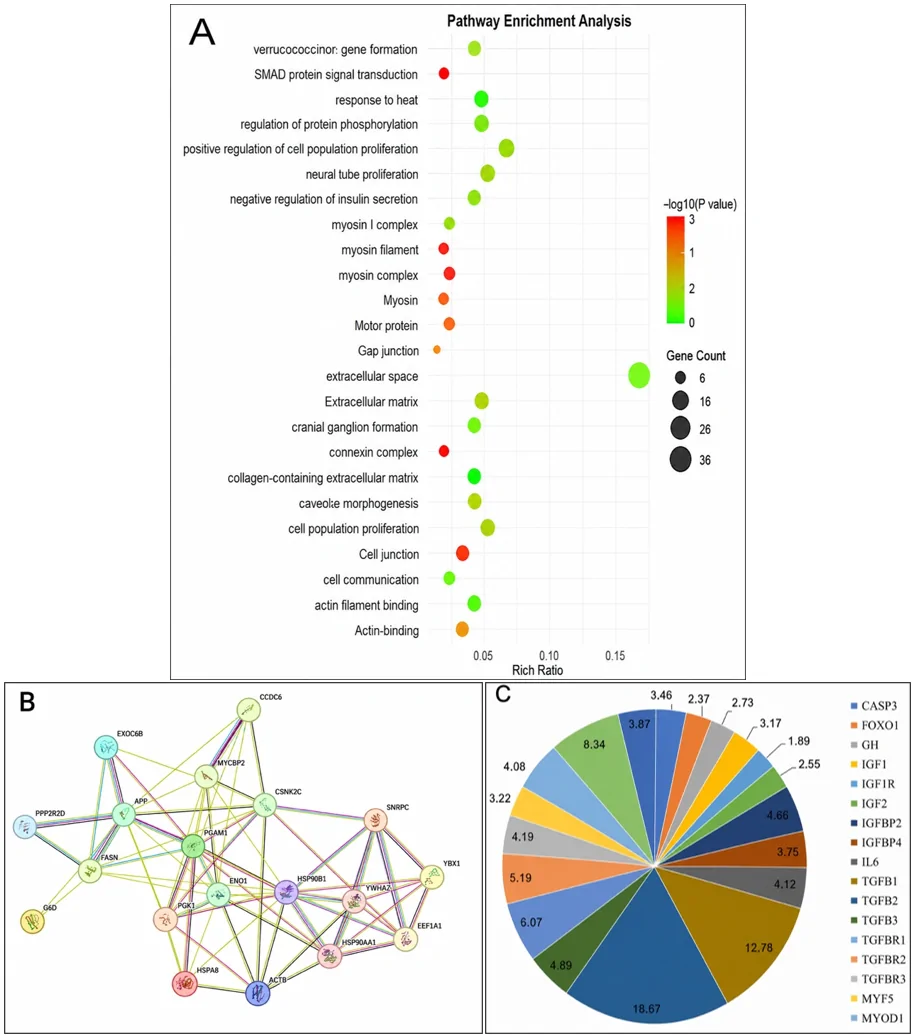
Functional characterization and interaction analysis of DEGs in KUB chickens. (A) Pathway enrichment analysis (http://www.bioinformatics.com.cn/). (B) The gene interaction of DEGs (https://cn.string-db.org/). (C) The Venn Plot of DEGs (http://www.bioinformatics.com.cn/).

Over 80% of the reads were successfully mapped to the reference genome of the chicken (*Gallus gallus*). After the mapping process, the expression level of each gene was calculated as the average, and genes showing significant DEGs in KUB chickens were identified (Figure 1A). The three biological replicates of KUB chickens showed very similar expression patterns, according to hierarchical clustering analysis, suggesting that the sequencing data were highly biologically reproducible. A volcano plot visualized the distribution of DEGs identified using DESeq2, with the X-axis representing changes in gene expression (log2 Fold Change) and the Y-axis indicating statistical significance (-log10 Adjusted p-value). Genes with significantly higher expression are indicated by red dots, whereas genes with significantly lower expression are indicated by blue dots (Figure 1B). Overall, these results indicate that KUB chickens have several candidate genes that warrant further exploration to elucidate the regulatory mechanisms of gene expression related to muscle tissue growth and development (Figure 1C).

### Effect of the *TGFβ2* gene on FI, FCR, and ADG in KUB chickens

The results in Table 1 show no significant difference between the three *TGFβ2* genotypes in improving the performance of KUB chickens. However, there was a significant difference (p < 0.05) in BW in the F4P7 treatment with the TC genotype, but no significant difference was found between the C and T genotypes. The dominant BW of KUB chickens with the CT genotype in the F4P7 treatment was 1586 g/bird, 1584 g/bird, and 1582 g/bird, respectively. The TC genotype in the F4P5 treatment was 1537 g/bird, followed by the CC genotype at 1535 g/bird and the TT genotype at 1532 g/bird. The increase in BW is suspected to be related to the KUB chicken genotype.

Table 1 also shows significant differences between the three *TGFβ2* genotypes in carcass weight (CW). Meanwhile, there was a significant difference in CW in the control treatment genotypes, namely TT 848 g/head, 852 g/head, and 850 g/head. The F4P7 treatment with the dominant TC genotype was higher at 1198 g/head, followed by the CC genotype at 1194 g/head, and then the TT genotype at 1190 g/head. The data obtained from this study are presented in Table 1. The *TGFβ2* genotype did not differ significantly in FI. However, in the F4P5 treatment, the CT genotype had a higher FI compared to the other treatments, namely 3881 g/head/90 days, followed by the CC genotype at 3880 g/head/90 days. In general, Table 1 shows a significant difference between the three *TGFβ2* genotypes in the FCR in the F4P3 treatment of TC genotypes 2.70, CC 2.72, and TT 2.76. While there was no significant difference with the F4P7 treatment, a high FCR was detected in the CC genotype control treatment, namely 4.21.

**Table 1:** The association between TGFβ2 genotype and FI, FCR, BW and CW in 3-months-Old.

**Treatment**	**Feed Intake (g/ekor/90 day)** **CC (n = 5)**	**FCR**					**BW ** **(g/ekor/90 day)** **CC (n = 5)**	**CW (g/ekor/90 day)**				
		**TC** **(n = 14)**	**TT** **(n = 6)**	**CC** **(n = 5)**	**TC** **(n = 14)**	**TT** **(n = 6)**		**TC** **(n = 14)**	**TT** **(n = 6)**	**CC** **(n = 5)**	**TC** **(n = 14)**	**TT** **(n = 6)**
F0P0	3872 ± 1.27	3871 ± 1.17	3870 ± 1.21	4.18^d^ ± 0.67	4.15^d^ ± 0.47	4.21^d^ ± 0.37	1142^d^ ± 1.96	1141^d^ ± 1.06	1143^d^ ± 1.11	850^d^ ± 2.61	852^d^ ± 1.91	848^d^ ± 2.01
F4P0	3873 ± 1.22	3873 ± 1.11	3874 ± 1.09	3.16^c^ ± 0.24	3.13^c^ ± 0.27	3.19^c^ ± 0.14	1438^c^ ± 1.85	1440^c^ ± 1.02	1436^c^ ± 1.15	1014^c^ ± 1.19	1018^c^ ± 1.24	1010^c^ ± 2.19
F4P3	3878 ± 1.77	3877 ± 1.10	3879 ± 1.12	2.73^a^ ± 0.35	2.70^a^ ± 0.36	2.76^a^ ± 0.17	1499^b^ ± 1.67	1502^b^ ± 1.06	1494^b^ ± 1.19	1118^c^ ± 1.53	1121^bc^ ± 1.59	1115^c^ ± 2.13
F4P5	3880 ± 1.21	3881 ± 1.12	3879 ± 1.11	2.84^a^ ± 0.16	2.85^a^ ± 0.22	2.86^a^ ± 0.19	1535^ab^ ± 1.20	1537^ab^ ± 1.03	1532^ab^ ± 1.13	1152^b^ ± 2.36	1154^b^ ± 1.76	1150^b^ ± 2.06
F4P7	3877 ± 1.13	3877 ± 1.15	3878 ± 1.15	2.94^b^ ± 0.15	2.92^b^ ± 0.29	2.96^b^ ± 0.12	1584^a^ ± 1.06	1586^a^ ± 1.07	1582^a^ ± 1.12	1194^a^ ± 2.20	1198^a^ ± 1.24	1190^a^ ± 2.09

Different superscripts (ᵃ^–^ᶜ) within the same column indicate significant differences (p < 0.05). Values are presented as mean ± SD. FI = Feed intake; FCR = Feed conversion ratio; BW = Body weight; CW = Carcass weight. Diets: F0P0 = Control (without phytase or protease supplementation); F4P0 = Phytase at 400 mg/kg without protease; F4P3 = Phytase at 400 mg/kg + protease at 300 mg/kg; F4P5 = Phytase at 400 mg/kg + protease at 500 mg/kg; F4P7 = Phytase at 400 mg/kg + protease at 700 mg/kg.

### Meat quality of KUB chicken

Dietary multi-enzyme affected various meat quality traits, such as pH, texture, CL, and WHC (Table 2), as well as color parameters (Table 3). Meat quality analysis showed that pH and texture effects were not significantly different (p > 0.05) between the multienzyme addition treatment and the treatment without multienzyme in the feed, but were significantly different (p < 0.05) in CL and WHC, as seen in Table 2. Color parameters were significantly higher (p < 0.05) in the multienzyme feed group compared to the control group, and there was a significant difference (p < 0.05) between the lower (F4P0) and higher (F4P3, F4P5 and F4P7) multienzyme concentrations; consequently, the results for other different analyses were based on the Lightness of KUB chicken meat. Redness of KUB chicken was significantly increased (p < 0.05) between the control group (F0P0) and the multienzyme treatments (F4P3, F4P5, and F4P7). The results of the analysis of the yellowness of KUB chicken meat showed a significant decrease (p < 0.05) in the F4P5 and F4P7 multienzyme groups when compared with the F4P0 and F4P3 groups; then, the group with the highest dose of multienzyme (F4P7) decreased significantly (p < 0.05) when compared with the control group (F0P0) without multienzymes in the feed.

**Table 2:** Meat quality traits (pH, texture, cooking loss, and water holding capacity) of KUB chickens with different TGFβ2 genotypes.

**Treatment**	**pH**			**Texture (N)**			**CL (%)**			**WHC (%)**			
	**CC** **(n = 5)**	**TC** **(n = 14)**	**TT** **(n = 6)**	**CC** **(n = 5)**	**TC** **(n = 14)**	**TT** **(n = 6)**	**CC** **(n = 5)**	**TC** **(n = 14)**	**TT** **(n = 6)**	**CC** **(n = 5)**	**TC** **(n = 14)**	**TT** **(n = 6)**
F0P0	5.87 ± 0.47	5.82 ± 0.72	5.77 ± 0.52	19.37^d^ ± 1.97	18.99^d^ ± 1.07	19.37^d^ ± 1.17	35.58^d^ ± 0.69	34.18^d^ ± 0.55	34.39^d^ ± 0.92	21.31^d^ ± 1.82	22.22^d^ ± 1.42	21.31^d^ ± 1.62
F4P0	5.61 ± 0.39	5.55 ± 0.59	5.91 ± 0.69	22.71^a^ ± 1.22	21.22^c^ ± 1.12	21.76^b^ ± 1.41	30.65^c^ ± 1.41	29.22^b^ ± 1.41	30.73^c^ ± 1.43	28.63^c^ ± 1.31	27.19 ± 1.11	28.33^c^ ± 1.17
F4P3	5.68 ± 0.26	5.72 ± 0.46	5.56 ± 0.36	22.40^a^ ± 1.48	22.63^a^ ± 1.09	21.35^b^ ± 1.11	29.66^b^ ± 1.72	29.54^b^ ± 1.72	29.72^b^ ± 1.63	28.78^c^ ± 1.19	29.29^b^ ± 1.09	28.48^c^ ± 1.21
F4P5	5.49 ± 0.31	5.81 ± 0.61	5.64 ± 0.51	21.39^b^ ± 1.15	21.18^c^ ± 1.02	21.77^b^ ± 1.10	28.82^a^ ± 1.54	28.78^a^ ± 1.54	29.52^b^ ± 1.34	29.98^a^ ± 1.12	29.47^b^ ± 1.16	29.55^b^ ± 1.19
F4P7	5.79 ± 0.22	5.49 ± 0.32	5.75 ± 0.42	21.52^b^ ± 1.25	21.68^b^ ± 1.14	21.49^b^ ± 1.21	28.74^a^ ± 1.76	28.55^a^ ± 1.76	28.99^b^ ± 1.66	29.98^a^ ± 1.14	29.63^a^ ± 1.13	29.77^a^ ± 1.16

^a-d^Means with varying superscripts within the same column differ significantly (p < 0.05) (Mean ± SD). CL = Cooking Loss, WHC = Water Holding Capacity. Diets: F0P0 = control (without phytase enzyme); F4P0 = Phytase enzyme at 400 mg/kg without protease; F4P3 = Phytase enzyme at 400 mg/kg + protease enzyme at 300 mg/kg; F4P5 = Phytase enzyme at 400 mg/kg + protease enzyme at 500 mg/kg; F4P7 = Phytase enzyme at 400 mg/kg + protease enzyme at 700 mg/kg.

**Table 3:** Meat quality traits and color attributes (L*, a*, and b*) of KUB chickens with different TGFβ2 genotypes.

**Treatment**	L*			a*			b*		
	**CC (n = 5)**	**TC (n = 14)**	**TT (n = 6)**	**CC (n = 5)**	**TC (n = 14)**	**TT (n = 6)**	**CC (n = 5)**	**TC (n = 14)**	**TT (n = 6)**
F0P0	43.73^d^ ± 2.09	42.66^d^ ± 2.09	40.62^d^ ± 2.19	4.13^d^ ± 1.72	4.73^d^ ± 1.22	4.99^d^ ± 1.12	14.15^d^ ± 1.41	14.62^d^ ± 1.11	14.55^d^ ± 1.01
F4P0	48.99^b^ ± 2.11	48.12^c^ ± 2.11	48.42^c^ ± 2.17	8.75^b^ ± 1.11	7.75^c^ ± 1.21	8.15^c^ ± 1.17	9.28^b^ ± 1.18	9.87^c^ ± 1.23	9.28^b^ ± 1.03
F4P3	49.25^b^ ± 2.12	49.57^a^ ± 2.12	48.29^b^ ± 2.32	8.19^b^ ± 1.71	9.25^a^ ± 1.32	8.19^c^ ± 1.11	9.05^b^ ± 1.21	9.55^c^ ± 1.24	8.73^a^ ± 1.11
F4P5	49.41^a^ ± 2.54	49.34^a^ ± 2.54	48.73^b^ ± 2.44	9.45^a^ ± 1.59	9.65^a^ ± 1.35	8.45^b^ ± 1.19	8.59^a^ ± 1.22	9.39^b^ ± 1.27	8.32^a^ ± 1.04
F4P7	49.57^a^ ± 2.54	49.14^b^ ± 2.54	49.77^a^ ± 2.14	9.27^a^ ± 1.43	9.99^a^ ± 1.32	8.87^b^ ± 1.13	8.09^a^ ± 1.27	9.01^b^ ± 1.09	8.17^a^ ± 1.06

^a-d^Means with varying superscripts within the same column differ significantly (p < 0.05) (Mean ± SD). L* = Lightness, a* = Redness, b* = Yellowness. Diets: F0P0 = control (without phytase enzyme); F4P0 = Phytase enzyme at 400 mg/kg without protease; F4P3 = Phytase enzyme at 400 mg/kg + protease enzyme at 300 mg/kg; F4P5 = Phytase enzyme at 400 mg/kg + protease enzyme at 500 mg/kg; F4P7 = Phytase enzyme at 400 mg/kg + protease enzyme at 700 mg/kg.

### Distribution of genotype and allele frequency

Table 4 presents the genotypic distribution of the *TGFβ2* gene coding region, as determined by distinct restriction patterns obtained from enzymatic digestion. The frequencies of the CC, TC, and TT genotypes were 20%, 56%, and 24%, respectively. Moreover, these genotype proportions showed highly significant differences (p > 0.05). According to the Hardy–Weinberg equilibrium, the allele frequencies for C and T were 0.48 and 0.52, respectively. Chi-square analysis revealed a strong association between the chicken strain and the AluI restriction enzyme. Such variations could be attributed to factors including gene flow across populations, interbreeding among groups, and differences in sample size.

### Genetic variation in the *TGFβ2* genes

**Figure 2:**
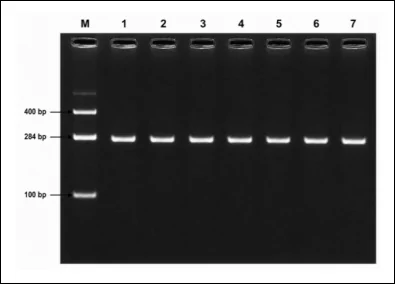
Electrophoresis of the PCR amplified *TGFβ2* gene showing bands of approximately 284 bp on 1.5% agarose gel. M = 100 bp DNA ladder.

**Figure 3:**
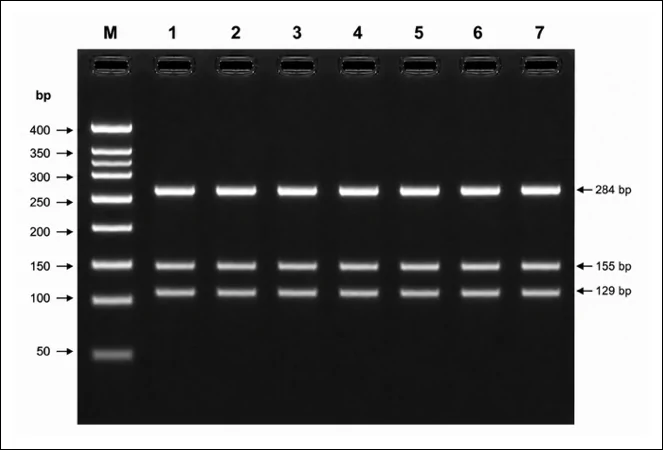
Alul restriction fragment patterns of *TGFβ2* by PCR-RFLP on 1.5% agarose gel; marker 284 pb, 155 bp, 129 bp; CC/TT/CT = genotype.

**Figure 4:**
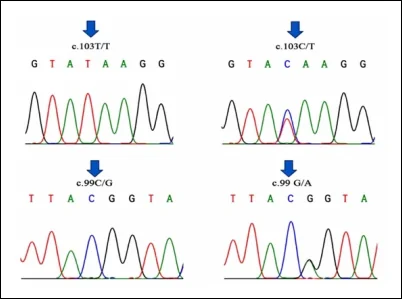
Partial nucleotide sequence of the *TGFβ2* gene in KUB chickens obtained by DNA sequencing.

Amplification was performed to identify SNPs by DNA sequencing (Figure 2). SNPs were detected using the F4P3 primer pair, whereas no polymorphisms were observed with the *TGFβ2* primer pair (Figure 3). Two SNPs were identified within the exon 1 region (c.103C/T and c.99G/A). The g.1079C/T substitution altered the codon from GGC to GGT, representing a synonymous mutation that still encodes glycine (Figure 4). DNA sequencing successfully genotyped the c.103C/T and c.99G/A SNPs, with the g.103C/T locus exhibiting CC and CT genotypes, while the g.99G/A locus showed GG and GA genotypes.

**Table 4:** Distribution of TGFβ2 genotype and allele frequency in KUB chickens

**Genotype**	**Number**	**Percentage (%)**
CC (Wild)	5	20
TC (Heterozygous)	14	56
TT (Mutant)	6	24
Total	25	100

χ² = 0.37 (p > 0.05). Allele frequencies: C: 0.48, T: 0.52.

In this study, two SNPs were identified for the *TGFβ2* gene target (Figure 3). SNP identification was performed by comparing the KUB chicken sequence and two local chicken sequences published in NCBI (Acc. X58071). Consequently, two SNPs were identified in the *TGFβ2* gene target. One SNP was detected in the F4P5 treatment, and another in the F4P7 treatment (Figure 4). However, these SNPs were not confirmed among the F0P0, F4P0, and F4P3 samples. In fact, two other SNPs were found in the same gene target (Figure 3).

## DISCUSSION

### Functional and pathway enrichment analysis of target genes

Previous research has identified several genes and signaling pathways that play key roles in muscle fiber formation and pectoral muscle growth across various chicken breeds [14]. However, molecular mechanisms underlying muscle development in the selectively bred KUB chicken remain insufficiently explored. The results of this study indicate that DEGs exhibit considerable variation in expression across developmental stages. GO enrichment analysis indicates that most DEGs are involved in biological processes related to muscle tissue growth, cell differentiation, and contractile function [15]. Furthermore, significant differences in *TGFβ2* gene expression levels were observed in KUB chickens, suggesting that this gene exhibits a specific regulatory pattern in muscle development.

Importantly, the KEGG and GO enrichment analyses of DEGs showed notable enrichment in several biological pathways, including focal adhesion, extracellular matrix (ECM)–receptor interaction, and cysteine and methionine metabolism, all of which support increased metabolic activity and muscle tissue development in KUB. Phytase improves phosphorus bioavailability, while protease enhances amino acid digestibility, both of which are critical for protein synthesis and satellite cell activity. Enhanced nutrient supply may therefore potentiate *TGFβ2*-regulated muscle development pathways, particularly in specific genotypes [16]. The observed genotype-enzyme-level interaction indicates that the growth response to multienzyme supplementation varies with *TGFβ2* genotype, supporting the presence of a nutrigenetic mechanism in KUB chickens [17]. This integrative gene-nutrition interaction highlights the potential of *TGFβ2* as a candidate marker for genetic selection and for precision feeding strategies in indigenous-derived tropical poultry systems [18].

### Effect of the *TGFβ2* gene on FI, FCR, BW, and BW gain in KUB chickens

Physiologically, improved growth performance in livestock is highly dependent on genetic activity that regulates the proliferation, differentiation, and maturation of skeletal muscle cells. One key gene that coordinates these processes is *TGFβ2* [19]. This gene directly affects growth efficiency and production performance by modulating cell repair processes and preserving the delicate balance between growth and tissue regeneration [20]. Furthermore, *TGFβ2* is involved in regulating physiological processes related to nutrient utilization and energy homeostasis, thereby indirectly influencing FI and energy conversion in chickens.

Research shows that genotypic variation in the *TGFβ2* gene is significantly associated with differences in BW gain and final weight in chickens, indicating a genetic link between the gene’s expression pattern and growth performance [21]. Individuals with genotypes that express *TGFβ2* more actively tend to exhibit higher growth efficiency without significant increases in feed consumption, as well as better physiological resistance to stress and disease [22]. These findings suggest that *TGFβ2* may contribute to improved feed conversion efficiency by optimizing muscle growth metabolism. Therefore, chickens with elevated *TGFβ2* expression may possess superior genetic merit for growth performance and overall productivity [23].

### Meat quality

The influence of multienzyme supplementation on meat physicochemical properties, including pH, texture, CL, and water holding capacity (WHC) as presented in Table 2 and on meat color parameters such as lightness (L*), redness (a*), and yellowness (b*) as shown in Table 3, was systematically evaluated. The pH value is a critical determinant of meat quality, as it directly affects protein denaturation, CL, and textural characteristics. A negative correlation was observed between pH and both lightness (L*) and yellowness (b*), whereas redness (a*) exhibited a positive correlation with pH. Analysis of breast meat from chickens fed multienzyme-supplemented diets demonstrated a decrease in yellowness (b*) and a corresponding increase in redness (a*), suggesting improved meat coloration. The correlation coefficients between color attributes (L*, a*, b*) and pH were highly significant, indicating a strong relationship between enzymatic treatment and color stability. Changes in meat pH and texture are often linked to microbial contamination, such as *Salmonella* infection, which can decrease pH and compromise texture [24]. A lower pH, associated with higher redness (a*) values, may also influence consumer perception of freshness and overall meat quality, as optimal chicken meat typically exhibits enhanced lightness and redness [25].

CL value decreased significantly as multienzyme dosage increased, whereas both texture and WHC improved markedly (Table 2). This finding is consistent with previous reports indicating that multienzyme supplementation enhances WHC by promoting improved moisture retention within muscle tissues [26]. CL was calculated as the percentage difference between raw and cooked sample weights and was lowest in the SRC group, which also exhibited the highest WHC. This suggests that multienzyme supplementation may enhance meat juiciness by reducing water loss during cooking [27]. The improvement may be attributed to the higher multienzyme concentration in the feed, which facilitates the formation of a denser, more cohesive muscle structure [28]. Furthermore, the type and dosage of multienzymes significantly affected meat texture, likely through enzymatic and biochemical modifications influencing muscle pigment oxidation and structural protein integrity. These changes were associated with improved color characteristics and textural quality [26]. Correlation matrix analysis revealed that CL was inversely related to texture parameters, including chewiness and firmness, suggesting a potential interaction between structural integrity and moisture retention capacity [27].

### Distribution of genotype and allele frequency

Crossbreeding is an important mechanism that contributes to increased genetic diversity and heterozygosity in livestock populations [29]. The process of gene flow between populations introduces new alleles into the gene pool, thereby enriching genetic variation and increasing heterozygosity at both the population and subpopulation levels [30]. In the context of the *TGFβ2* gene, previous studies have reported two major alleles (T and C) and three genotype combinations (TT, CT, and CC) in a chicken population [31]. A similar polymorphism pattern was also found in local Indonesian chickens, including KUB chickens, with two alleles (T and C) and three genotypes (TT, CT, and CC) [32]. However, the frequency distribution showed dominance of the T allele over the C allele, likely influenced by inbreeding practices and strong selection pressure on the *TGFβ2* gene. Changes in the frequency of the *TGFβ2* allele in KUB chickens may reflect an adaptive response to environmental conditions and nutritional factors provided during rearing [33]. Feed supplementation with enzymes such as phytase and protease has been reported to affect the bioavailability of essential amino acids and minerals, thereby altering the transcriptional activity and expression of the *TGFβ2* gene [34].

Certain alleles of the *TGFβ2* gene, such as the T allele, are thought to be associated with higher nutrient utilization efficiency and increased muscle protein deposition, while the C allele may be associated with a more conservative metabolic strategy under conditions of nutrient deficiency or excess [35]. Thus, nutritional selection pressure arising from variations in feed composition can influence allele frequency dynamics in the KUB chicken population. These results indicate a synergistic interaction between genetic factors (*TGFβ2* gene polymorphisms) and nutritional factors (enzyme content and nutrient availability), which collectively contribute to metabolic adaptation, growth efficiency, and the physiological performance of KUB chickens in tropical environments.

### Genetic variation in the *TGFβ2* genes

The hypothalamus, which primarily expresses the *TGFβ2* gene, is crucial for regulating poultry FI and energy balance. Furthermore, this gene is expressed in other tissues, including erectile tissue, where *TGFβ2* is reported to contribute to physiological mechanisms underlying erectile function [36]. The candidate gene approach has been widely used to identify genomic regions associated with important traits in livestock, including specific genetic markers for selection and breeding. Several previous studies reported the presence of four variations in the *TGFβ2* gene promoter region in chickens [37], as well as five SNPs in the 5’UTR region and one SNP in the exon region (c.129A/G) [38]. Another study also identified four additional SNPs in the 3’UTR region using PCR-SSCP and DNA sequencing. In this study, analysis of a 387-bp fragment of the *TGFβ2* gene in KUB chickens revealed no significant polymorphisms. However, when the gene sequence was compared with GenBank (NCBI) reference data, two new SNPs were identified in the 5'UTR region. This difference indicates that the *TGFβ2* gene in KUB chickens has unique sequence variations compared with other poultry species, and thus could be used as a molecular marker for the genetic identification of KUB chickens.

Furthermore, this study also identified two SNPs in the exon region, as well as one additional SNP detected after comparison with the GenBank *TGFβ2* sequence. The two identified point mutations, c.103C/T and c.99G/A, are unique variations not found in the other two reference sequences in GenBank. This finding aligns with a previous report that also identified SNPs in the exon region of the *TGFβ2* gene in chickens [38]. That study reported one synonymous substitution at position c.93G/A (GG→AG) and one nonsynonymous substitution at position 293G/A, which causes an amino acid change from glycine to arginine. Overall, these results strengthen the evidence of genetic diversity in *TGFβ2* in KUB chickens and provide an important scientific basis for further research on the function of this gene in the physiological performance of local poultry. In addition, three restriction enzymes, RsaI, AluI, and KpnI, were successfully used for genotyping by PCR-RFLP [39]. These three enzymes recognize specific sequences GT’AC, G’GTAC_C, and G_GTAC’C, respectively. The use of restriction enzymes as a molecular analysis tool has proven effective for evaluating genetic diversity and gene function *in vivo*, and similar methods have also been applied in genotyping the *TGFβ2* gene in other local chicken populations [40].

## CONCLUSION

This study provides the first comprehensive characterization of *TGFβ2* gene polymorphisms in KUB chickens and demonstrates their association with growth performance and meat quality in response to multienzyme supplementation. Three *TGFβ2* genotypes (CC, TC, and TT) were identified, with allele frequencies of 0.48 (C) and 0.52 (T). Significant associations between genotype and BW, CW, and FCR indicate that *TGFβ2* contributes to growth efficiency in this indigenous breed. In addition, dietary phytase and protease supplementation improved meat quality by reducing CL, increasing WHC, and enhancing meat color characteristics, while two novel exon SNPs (c.103C>T and c.99G>A) were identified in KUB chickens. Functional enrichment analyses further implicated extracellular matrix-receptor interaction, metabolic pathways, and muscle development processes, supporting the biological relevance of *TGFβ2* in regulating productive traits.

From a practical perspective, these findings provide valuable molecular evidence for integrating *TGFβ2* polymorphisms into MAS programs and highlight the potential of combining genetic selection with precision nutritional strategies to improve growth performance and meat quality in indigenous tropical chicken populations. Such an integrated approach could enhance production efficiency while preserving the desirable adaptive characteristics of KUB chickens, thereby contributing to sustainable poultry production and food security in tropical production systems.

A major strength of this study is the integration of molecular genotyping, growth performance evaluation, meat quality assessment, and bioinformatics analyses within a nutrigenetic framework, providing a comprehensive understanding of genotype–nutrition interactions. However, the study is limited by the relatively small sample size, evaluation of a single indigenous breed, and the absence of functional validation of the identified polymorphisms or direct gene expression analyses across genotypes. These limitations restrict the generalizability of the findings and preclude definitive conclusions regarding the biological mechanisms underlying the observed associations.

Future studies should validate these associations in larger, genetically diverse chicken populations, investigate the functional effects of the identified polymorphisms using transcriptomic and proteomic approaches, and evaluate genotype-specific nutritional responses under commercial production conditions. Such investigations will facilitate the development of genomic selection tools and precision feeding strategies for indigenous poultry breeding programs.

Overall, *TGFβ2* represents a promising candidate gene for improving growth performance and meat quality in KUB chickens. The findings establish an important foundation for future genomic breeding initiatives and support the application of molecular genetics and precision nutrition to enhance the productivity, sustainability, and competitiveness of indigenous poultry production systems.

## DATA AVAILABILITY

The supplementary data supporting the findings of this study are available from the corresponding author upon reasonable request.

## GENERATIVE AI DECLARATION

The authors declare that generative artificial intelligence (AI) tools were used solely to improve language, grammar, and readability during manuscript preparation. All scientific content, data analysis, interpretation of results, and conclusions were developed and verified by the authors. The authors take full responsibility for the accuracy, integrity, and originality of the work presented, and no AI tool was listed as an author.

## AUTHORS’ CONTRIBUTIONS

SRA: Conceptualized the study, conducted the experiment, performed molecular and laboratory analyses, analyzed the data, interpreted the results, and drafted the manuscript. ML: Conceived and supervised the study, interpreted the findings, and critically revised the manuscript. SHW and MAAA: Designed the experimental methodology, analyzed and interpreted the data, and critically edited the manuscript. EJK and SS: Contributed to data interpretation, critically reviewed the scientific content, and revised the manuscript. LL: Assisted with molecular data interpretation, reviewed the manuscript, and edited the references. ARK: Assisted with literature review, reference management, and manuscript editing. All authors have read and approved the final manuscript.

## ACKNOWELDGMENTS

The authors gratefully acknowledge the financial support provided by the Indonesian Education Scholarship (BPI), the Center for Higher Education Funding and Assessment (PPAPT), and the Indonesian Endowment Fund for Education (LPDP), Ministry of Higher Education, Science, and Technology of the Republic of Indonesia, under Grant No. 01366/BPPT/BPI.06/9/2023.
